# Identification of Emerging Industrial Biotechnology Chassis *Vibrio natriegens* as a Novel High Salt-Tolerant and Feedstock Flexibility Electroactive Microorganism for Microbial Fuel Cell

**DOI:** 10.3390/microorganisms11020490

**Published:** 2023-02-16

**Authors:** Zhijin Gong, Rong Xie, Yang Zhang, Meng Wang, Tianwei Tan

**Affiliations:** 1 National Energy R&D Center for Biorefinery, College of Life Science and Technology, Beijing University of Chemical Technology, Beijing 100029, China; 2 Beijing Key Laboratory of Bioprocess, College of Life Science and Technology, Beijing University of Chemical Technology, Beijing 100029, China

**Keywords:** *Vibrio natriegens*, bioelectricity, microbial fuel cell, feedstock flexibility, high salt resistance, biosynthesis of phenazine electron mediators

## Abstract

The development of MFC using electroactive industrial microorganisms has seen a surge of interest because of the co-generation for bioproduct and electricity production. *Vibrio natriegens* as a promising next-generation industrial microorganism chassis and its application for microbial fuel cells (MFC) was first studied. Mediated electron transfer was found in *V. natriegens* MFC (VMFC), but *V. natriegens* cannot secrete sufficient electron mediators to transfer electrons to the anode. All seven electron mediators supplemented are capable of improving the electronic transfer efficiency of VMFC. The media and carbon sources switching study reveals that VMFCs have excellent bioelectricity generation performance with feedstock flexibility and high salt-tolerance. Among them, 1% glycerol as the sole carbon source produced the highest power density of 111.9 ± 6.7 mW/cm^2^. The insight of the endogenous electronic mediators found that phenazine-1-carboxamide, phenazine-1-carboxylic acid, and 1-hydroxyphenazine are synthesized by *V. natriegens* via the shikimate pathway and the phenazine synthesis and modification pathways. This work provides the first proof for emerging industrial biotechnology chassis *V. natriegens* as a novel high salt-tolerant and feedstock flexibility electroactive microorganism for MFC, and giving insight into the endogenous electron mediator biosynthesis of VMFC, paving the way for the application of *V. natriegens* in MFC and even microbial electrofermentation (EF).

## 1. Introduction

More voices are calling for a quicker transition towards clean energy with the increasing importance of environmental protection and reducing carbon emissions [1,2]. Microbial fuel cells (MFC) have been considered a promising alternative system to traditional power sources as they can convert the chemical energy stored in organic substrates, fermentation products, and sewage into electrical energy without generating pollutants by electroactive microorganisms [3]. In the MFC system, electroactive microorganisms play the role of biocatalysts and can directly or indirectly transfer electrons obtained from the oxidation of organic compounds to the anode to generate currents [4], which is vital for the start-up of MFC. At present, the isolated electroactive microorganisms are mainly distributed in *Shewanella* and *Geobacter* [5]. Moreover, microorganisms such as *Pseudomonas aeruginosa* [6,7], *Klebsiella sp.* [8], *Escherichia coli* [9,10], *Saccharomyces cerevisiae* [11,12], *Arcobacter butzleri* [13], and *Bacillus subtilis* [14] have also been applied in MFC. Among them, industrial microorganisms such as *Escherichia coli* and *Saccharomyces cerevisiae* are favored owing to their genetic tractability and multi-purpose application potentiality in producing bioelectricity and fermentation products simultaneously [15]. For instance, Feng et al. [9] reported the stable output voltages of *E. coli*-inoculated MFCs are increased more than twice by introducing a heterologous phenazine-1-carboxylic acid pathway. Yong et al. [16] reported disrupting the gene *ldhA* encoded Lactate dehydrogenase; the maximum current density of *E. coli* BL21 strain is increased by approximately five times. Yuan et al. [17] reported that a co-generation system of bioethanol and electricity using a yeast MFC could be maintained, giving a stable voltage of around 350 mV and an ethanol yield of around 90% of the theoretical yield in 96 h. In addition, some industrial or potential industrial microorganisms that can be applied to MFC, such as *E. coli* and *Klebsiella pneumoniae*, are also able to be applied in electrochemistry (EF) by neutral red-mediated inward the extracellular electron transfer to improve fermentation production [18,19,20]. Therefore, the development of MFCs using industrial or potential industrial microorganisms has seen a surge of interest.

High salinity wastewater refers to wastewater with a total dissolved inorganic salt content greater than 1% [21]. Many industries generate high-salinity wastewater with high organic strength, such as the fish industry, food processing, textile, leather, and petroleum industries. Seafood processing produces wastewater characterized by salinity between 30 and 50 g/L NaCl [21]. Textile-produced wastewater can contain as much as 50 to 60 g/L NaCl [22]. Nowadays, high-salinity wastewater with high organic strength is likely to represent up to 5% of the wastewater produced globally and is difficult and costly to treat [23]. MFC is a promising wastewater treatment system that can realize the transformation of wastewater treatment from energy consumption to energy generation and is expected to break through the dilemma of difficult and costly treatment of high-salinity wastewater [24]. However, the majority of the research concerning MFCs was performed in a condition of low salinity because when bacterial cells are in a solution characterized by high salinity concentrations, they can suffer dehydration, where water that exits the cellular membrane leads to cell death [21]. Therefore, screening of electroactive microorganisms with high salt-tolerant and feedstock flexibility is essential for the treatment of high salinity wastewater with high organic strength by MFC.

**Figure 1:**
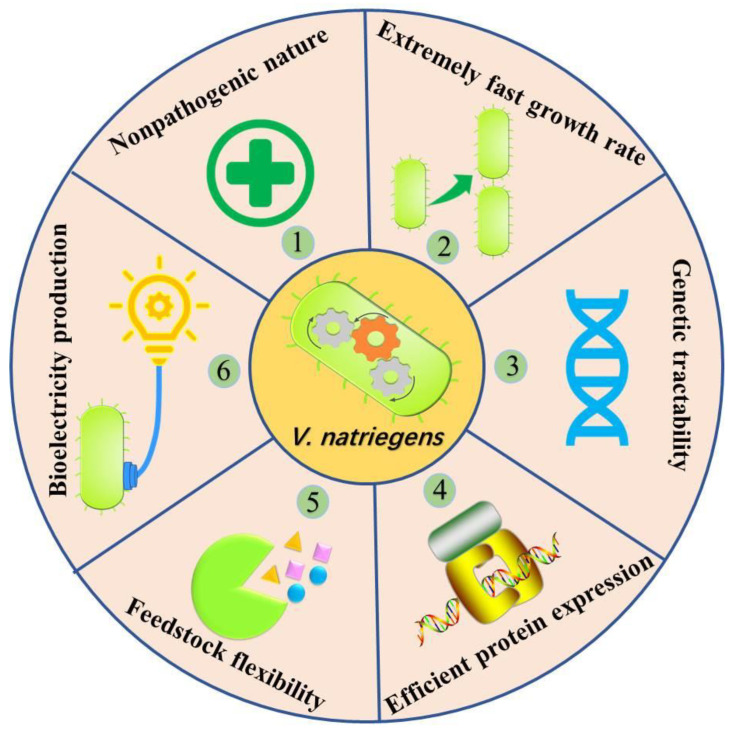
The inherent benefits of *V. natriegens*. Numbers 1 to 5 show the previously reported inherent benefits, and number 6 is the bioelectric production which is the inherent benefit described in this study.

*V. natriegens* is an emerging industrial biotechnology chassis, and the nonpathogenic nature [25], genetic tractability [26,27], high substrate uptake rates [28,29], remarkably short metabolic prowess, and efficient protein expression [30] of *V. natriegens* make it a promising next-generation industrial microorganism [25,26,31,32] (Figure 1). Genetically engineered *V. natriegens* strains have been used to produce diverse chemicals such as 1,3-propanediol [33], 2,3-butanediol [31,34], PHB [,^28^,^35^], lycopene [31], succinic acid [36], alanine [29], and ethanol [31]. Typically, targeting the expression of nine genes involved in PHB biosynthesis, the engineered *V. natriegens* strain produced 100-fold more PHB than the wild-type strain [35], and *V. natriegens* with knockout of *dldh*, *lldh*, *pfl,* and *mdh* genes produced alanine from glucose with a satisfying yield of 0.81 g/g [29]. In addition, according to previous reports, suitable fermentation media for *V. natriegens* generally contain 30 g/L sodium chloride (high salinity medium). Therefore, expanding the application of the emerging industrial biotechnology chassis *V. natriegens* to MFC may provide a novel high salt-tolerant electroactive industrial microorganism for the development of MFC and even EF. However, to the best of our knowledge, no studies have been implemented in this area to date, which restricts the development of *V. natriegens* in MFC and even EF.

**Figure 2:**
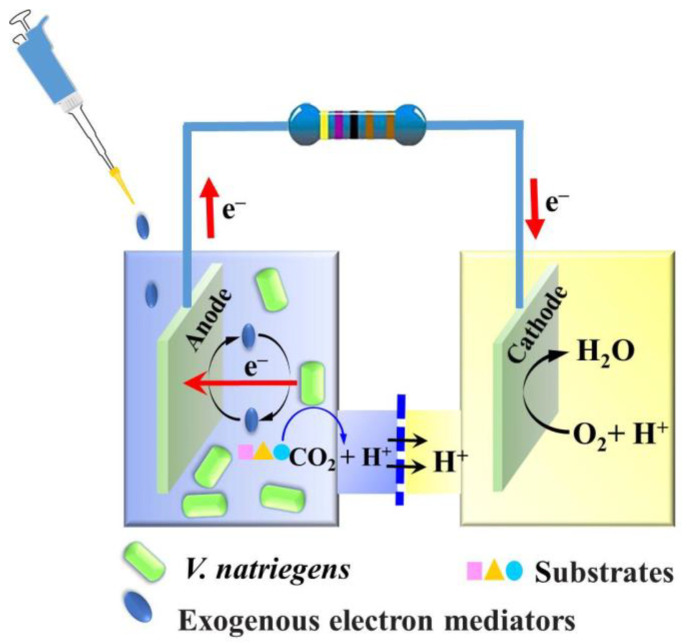
Schematic representation of *V. natriegens* MFC used in this study.

In this study, we are the first to apply *V. natriegens* to MFC (Figure 2) and show that *V. natriegens* is a promising high salt-tolerant and feedstock flexibility electricity-producing microorganism for MFC (Figure 1), meanwhile giving insight into the biosynthesis of endogenous electron mediators of *V. natriegens* for MFC. Although this research is a conventional evaluation and identification study of MFC, its results are of great value as a baseline for meaningfully expanding the application of *V. natriegens* to advanced MFC and even EF in the future.

## 2. Materials and Methods

### 2.1. Culture Media and Reagents

Lysogeny broth 3 (LB3) medium was prepared by adding 20 g/L NaCl to Lysogeny broth (LB) for a total of 30 g/L of NaCl. The M9 medium is composed of M9 minimal medium, 10 g/L glucose, and 22.5 g/L NaCl (an additional 20 g/L NaCl was also added except for the 2.25 g/L NaCl contained in the M9 minimal medium). Among them, the M9 minimal medium is composed of the following (1 L): Na_2_HPO_4_ 34.0 g, KH_2_PO_4_ 15.0 g, NaCl 2.5g, and NH_4_Cl 5.0 g. The VN medium (modified CGXII medium) is composed of the following (1 L) [29]: 10 g glucose, 5 g (NH_4_)_2_SO_4_, 15 g NaCl, 1 g KH_2_PO_4_, 1 g K_2_HPO_4_, 0.25 g MgSO_4_, 0.01 g CaCl_2_, 16.4 mg FeSO_4_·7H_2_O, 10 mg MnSO_4_·H_2_O, 0.3 mg CuSO_4_·5H_2_O, 1 mg ZnSO_4_·7H_2_O, and 0.02 mg NiCl_2_·6H_2_O. The 2661 medium contains 10 g/L glucose, and the 2661E minimal medium is provided by China General Microbiological Culture Collection Center (CGMCC) for culturing *V. natriegens*. Among them, the 2661E minimal medium is composed of the following (1 L): peptone 5.0 g, yeast extract 1.0 g, FeC_6_H_5_O_7_ 0.1 g, NaCl 19.45 g, MgCl_2_ 5.9 g, MgSO_4_ 3.24 g, CaCl_2_ 1.8 g, MgCl_2_ 0.55 g, NaHCO_3_ 0.16 g, KIO_4_ 0.08 g, SrCl_2_ 34.0 mg, H_3_BO_3_ 22.0 mg, Na_2_SiO_3_·9HO_2_ 4.0 mg, NaF 2.4 mg, NH_4_NO_3_ 1.6 mg, and Na_2_HPO_4_ 8 mg. The YPD medium is composed of the following (1 L): yeast extract 10 g, peptone 20.0 g, glucose 20 g. In addition, in this study, general reagents and chemicals were purchased from Macklin Inc (Shanghai, China), their purity is analysis pure, and LB3 was used as seed medium for culturing *V. natriegens* unless otherwise stated.

### 2.2. MFC Setup and Operation

The dual-chamber MFC (80 mL for both anode and cathode chambers) were separated by Nafion 117 membrane (Gashub, Singapore), and carbon cloths were utilized as both the anode (3.0 cm × 3.0 cm) and the cathode (3.0 cm × 3.0 cm). Carbon cloths were soaked in 1 M HCl and acetone for 12 h, respectively, before being used. The anode contained 80 mL of medium, and the cathodic electrolyte contained 5% K_3_[Fe(CN)_6_] and 80 mL KH_2_PO_4_ solution. The KH_2_PO_4_ solution was adjusted to the same pH as the corresponding anode medium by adding 1 M NaOH before being used. Herein, the pH values of LBV3, VN, M9, and 2661 anode medium were 6.9, 6.3, 7.1, and 7.7, respectively.

### 2.3. Culture Condition

*V. natriegens* CGMCC 1.8729, from the China General Microbiological Culture Collection Center (CGMCC), was cultivated separately in LB3 medium at 30 °C, 200 rpm for 5 h with the inoculation volume of 2%. The culture was then centrifuged at 9000 rpm for 5 min. The cells were harvested and washed twice with the same medium employed for the anode and then were inoculated into MFC until the optical density of MFC at 600 nm reached 1.7. The MFC inoculated cells were purged with sterilized nitrogen gas to remove oxygen and then were incubated at 37 °C and 300 rpm with a 1 kΩ external resistor. For reducing the effect of ME on cell absorbance, the relative *OD*_600_ was measured for calculation of the cell growth rate as follows: 0.2 mL of the anode culture solution was centrifuged at 12,000 rpm for 1 min, the supernatant was discarded, the cells were resuspended with 2 mL of distilled water, and then the cells were centrifuged again at 12,000 rpm for 1 min and was resuspended with 2 mL of distilled water for the absorbance measurement at 600 nm. The external circuit voltage and open circuit voltage were recorded by data acquisition cards CHI660E (Beijing Chinese science days Technology Co., Ltd., Shanghai, China). The external circuit current was recorded by a digital multimeter (VICTOR 890H, Victorlong Instruments, Shenzhen, China). The potentiostatic current of VMFC was also recorded by data acquisition cards CHI660E with the set potential of 100 mV and ample interval of 1 s. In addition, for running *E. coli* and *S. cerevisiae* MFC, the LB and YPD media were used in the anode chamber as the culture substrates, and the culture temperature was separately set at 37 °C and 30 °C. The measurement methods of cell growth rate, external circuit voltage, and open circuit voltage for *E. coli* MFC and *S. cerevisiae* are the same as for VMFC.

### 2.4. Characterization of Riboflavin, Phenazine-1-Carboxylic Acid, Phenazine-1-Carboxamide, and Hydroxyphenazine by LC-MS/MS

Analysis of riboflavin was performed on a Nexera XR HPLC series system (Shimadzu, Kyoto, Japan) coupled with a linear ion trap mass spectrometer as the detector (QTRAP 5500, AB Sciex Instruments, Framingham, MA, USA) with electrospray ionization (ESI) conditions in positive mode. The spray voltage was set to 4.5 kV for positive mode; the temperature was set to 550 °C; the declustering potential was 60 V; the collision energy was 15 eV; the scanning mode was set to selective reaction monitoring (MRM). The mobile phases solution used were methanol (Fisher Chemical Co., USA) and water with 0.1% (*v*/*v*) acetic acid (J&K Scientific, Beijing, China) = 60:40. An agilent eclipse plus C18 column (3.0 × 50 mm 1.8 μm; Agilent, Santa Clara, CA, USA) was used to chromatographically separate the riboflavin. The temperature of the column box was set to 40 °C, and the flow rate was set to 0.5 mL/min with a typical injection volume of 10 μL.

Analysis of phenazine-1-carboxylic acid (PCA), phenazine-1-carboxamide (PCM), and 1-hydroxyphenazine (HPZ) was performed on a SHIMADZU-LC20A, LCMS-8050 triple quadrupole LC/MS (Shimadzu, Kyoto, Japan) with electrospray ionization (ESI) conditions in positive mode. The interface temperature was 300 °C; interface voltage was 4.50 kV; drying gas flow rate was 10 mL/min; desolvation temperature was 526 °C; the scanning mode was set to MRM. The mobile phase solution used was acetonitrile (Fisher Chemical Co., Waltham, MA, USA) and water with 0.1% (*v*/*v*) acetic acid (J&K Scientific, Beijing, China). The chromatographic separation programs were performed as follows: 10% acetonitrile elution ran for 1 min, and increased to 85% from 1 to 5 min, then kept for 3 min at 85%, and then decreased to 10% from 8 to 8.5 min. A Waters ACQUITY UPLC BEH C18 column (100.0 × 2.1 mm 1.7 μm; Shimadzu, Kyoto, Japan) was used to chromatographically separate the PCA, PCM, and HPZ. The temperature of the column box was set to 40 °C, and the flow rate was set to 0.3 mL/min with a typical injection volume of 1 μL.

### 2.5. Data Analysis and Statistics

All MFC tested were built in three fully independent biological replicates, and the errors in these studies are the standard deviations of the three repeated. Potentiostat data analysis was performed using Origin 2021 (OriginLab Corporation, Northampton, MA, USA). The growth rate was calculated by linear regression of ln (*OD*_600_) plotted against time (in hours) during the exponential phase. The linear fit of ohmic loss in the polarization curve used the built-in linear fit method of Origin 2021. For LC-MS/MS analysis, three technical replicates were prepared for calculations of standard deviation.

## 3. Results

### 3.1. Evaluating the Bioelectricity Production Capacity of V. natriegens for MFC without Adding Exogenous Electron Mediators

**Figure 3:**
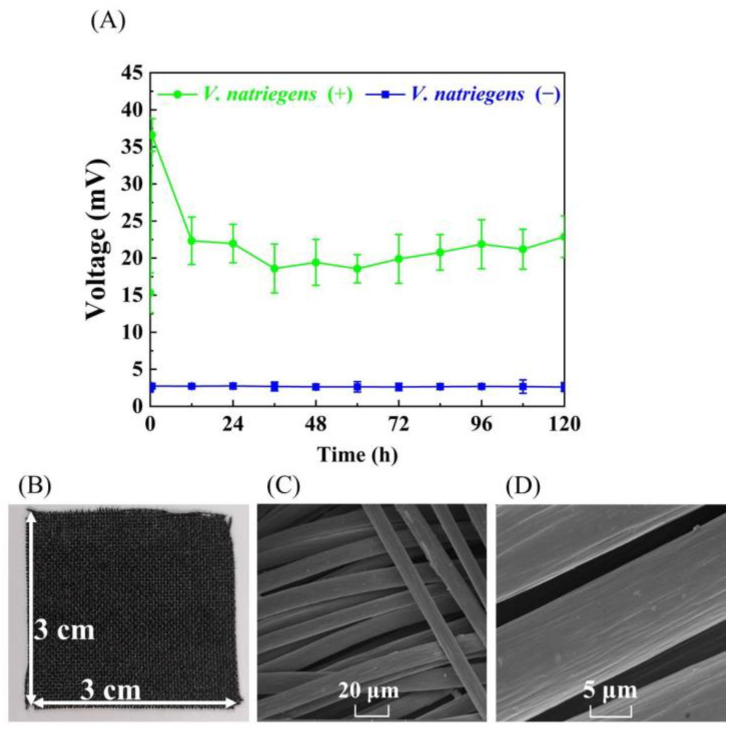
Evaluating the bioelectricity production capacity of *V. natriegens* by MFC without adding exogenous electron mediators. (**A**) External circuit voltage of LB3 MFC inoculated and uninoculated with *V. natriegens* (control group). The anode and cathode electrodes were connected through an external resistance of 1 kΩ. (+): inoculated with *V. natriegens*; (−): uninoculated with *V. natriegens*. (**B**) photo of carbon cloth anode from *V. natriegens* LB3 MFC running for 120 h; (**C**) SEM photos of carbon cloth anode with magnification of 2000 times and (**D**) SEM photos of carbon cloth anode with magnification of 10,000 times using a TESCAN MAIA3 scanning electron microscope (SEM) (TESCAN, Brno, Czech Republic).

A dual chamber MFC filled with 80 mL LB3 medium [26] in an anode chamber and a 1 kΩ external resistor was run for approximately 120 h to evaluate the bioelectricity production capacity of *V. natriegens.* As shown in Figure 3A, MFC inoculated with *V. natriegens* delivered an external circuit voltage between 20 and 30 mV, but MFC uninoculated with *V. natriegens* (control group) only produced external circuit voltages of 2.6 to 3.3 mV, indicating *V. natriegens* is unable to generate bioelectricity by MFC. In addition, no biofilm formation or cell adhesion was found on the surface of the carbon cloth anode after the end of culture for 120 h (Figure 3B–D). In light of this, we inferred that the extracellular electron transfers of *V. natriegens* MFC (VMFC) in this study are mediated electron transfers, and the low external circuit voltage delivered may be due to the inadequate electron mediators secreted by *V. natriegens*. Therefore, in the next experiments, we tried to add exogenous electron mediators to improve the electron transfer capability of VMFC.

### 3.2. Effects of Adding Exogenous Electron Mediators on the Bioelectricity Production Capacity of VMFC

**Figure 4:**
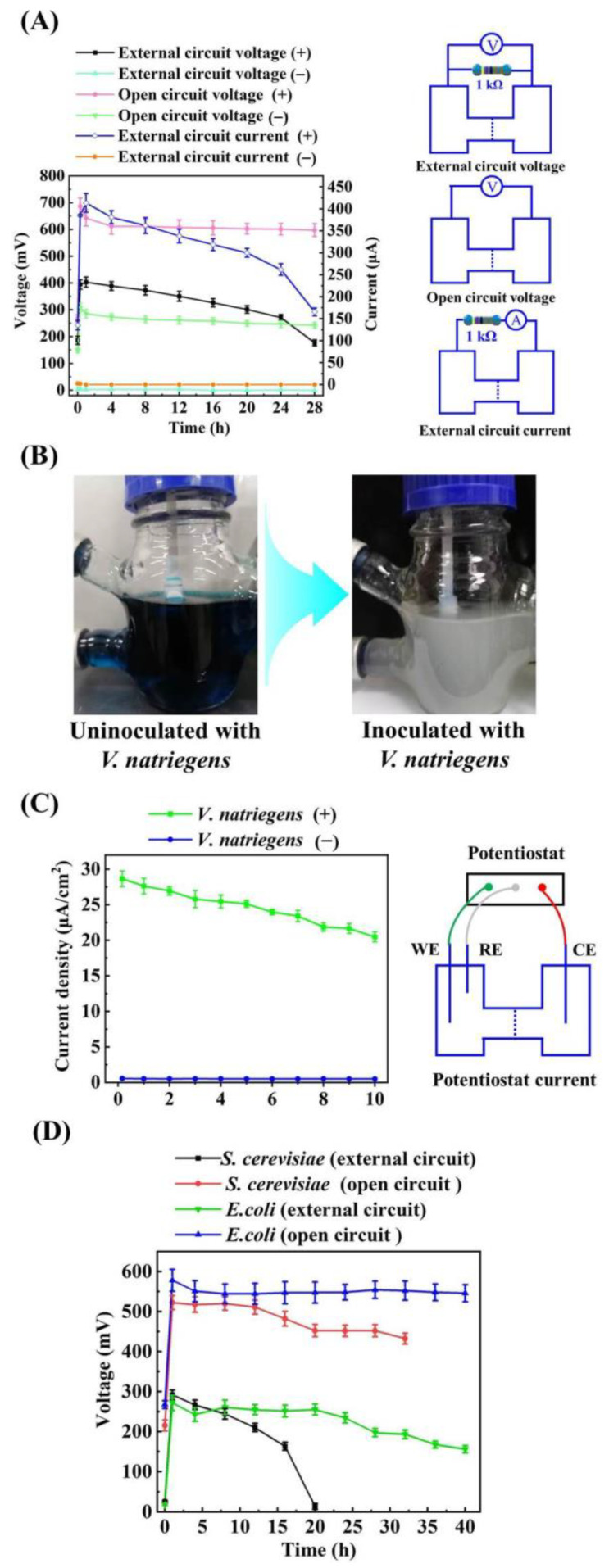
Bioelectricity generation of MFC with methylene blue as exogenous electron mediator. (**A**) The external circuit voltage, open circuit voltage, external circuit current, and corresponding circuit schematic of LB3 MFC inoculated and uninoculated with *V. natriegens*. (**B**) The digital images of anode inoculated and uninoculated with *V. natriegens* in the LB3 MFC. (**C**) The potentiostatic current density and circuit schematic of LB3 MFC inoculated and uninoculated with *V. natriegens*. (**D**) The external circuit voltage and open circuit voltage of *E. coli* MFC and *S. cerevisiae* MFC.

Next, we tested the bioelectricity production capacity of VMFC using LB3 medium (LB3 VMFC) supplemented with 0.4% methylene blue (MB), a commonly used non-physiological electron mediator [37]. As shown in Figure 4A, the external circuit voltage of VMFC with an external resistance of 1 kΩ exceeded 300 mV in 20 h, and the maximum external circuit voltage, external circuit current, open circuit voltage, and output power density reached 404.9 ± 21.6 mV, 413.7 μA, 702.6 ± 19.4 mV, and 91.1 ± 4.5 mW/cm^2^, respectively. The MB electron mediator at the anode changes from an oxidized state (blue) to a reduced state (colorless) when the *V. natriegens* is inoculated into the MFC (Figure 4B) and the growth rate of *V. natriegens* reached 0.08 h^−1^ (Figure S1). In addition, to further demonstrate that *V. natriegens* is electroactive and can produce bioelectricity, the potentiostatic current of MFC supplemented with 0.4% MB was measured by data acquisition cards CHI660E with the set potential of 100 mV and ample interval of 1 s. As shown in Figure 4C, the potentiostatic current of MFC inoculated with *V. natriegens* reached 368.4 μA to 515.7 μA in 10 h, while the MFC uninoculated with *V. natriegens* only delivered a potentiostatic current of 8.9 μA to 9.7 μA. These results suggested that *V. natriegens* is electroactive and can produce a considerable number of electrons to generate bioelectricity cultured in MFC; the extracellular electron transfer of VMFC is mediated electron transfer, but *V. natriegens* cannot synthesize enough electron mediators to transfer these electrons and requires exogenous supplementation of electron mediators. In addition, as a promising next-generation industrial microorganism chassis, the external circuit voltage of VMFC was compared with other MFCs used industrial microorganisms such as *E. coli* and *S. cerevisiae* to evaluate its bioelectricity generation capacity. As shown in Figure 4D, the *E. coli* MFC delivered the highest external circuit voltages of 271.94 mV and highest open circuit voltages of 578.3 mV, and the *S. cerevisiae* MFC delivered the highest external circuit voltages of 292.1 mV and highest open circuit voltages of 522.5 mV, which is lower than that of the VMFC (Figure 4A).

Furthermore, in order to assess whether other commonly used electron mediators can also enhance the efficiency of extracellular electron transfer in VMFCs, the LB3 VMFC with six other electron mediators, including four physiological electron mediators (riboflavin [38,39]; PCA [40]; phenazine-1-carboxamide, PCM [41]; 1-hydroxyphenazine, HPZ [42]) and two non-physiological electron mediators (disodium anthraquinone-2,6-disulfonate, DAD [43,44]; humic acid, HA [45]), were tested, respectively. The output power density of MFCs was obtained with a 1 kΩ external resistor. As shown in Table 1, all seven electronic mediators tested were able to enhance the output power density of MFCs. Among them, in the MFCs with physiological, the maximum power density delivered reached 8.0 ± 0.2 mW/cm^2^ with the addition of 20 mg/L PCA. In the MFCs with non-physiological electron mediators, the addition of 100 μmol/L DAD and 1.3 g/L humic acid (HA) delivered a maximum power density of 1.8 ± 0.1 mW/cm^2^ and 2.3 ± 0.1 mW/cm^2^, which is significantly lower than the output powers generated by adding MB. In addition, the external circuit voltages used above redox mediators without inoculated microorganisms (control experiments) were also tested in this study, but bare voltages were detected in all control experiments. Those results revealed that the extracellular electron transfer efficiency of VMFCs is unable to be improved by adding different electron mediators.

### 3.3. The Performance Evaluation of VMFCs with Media and Carbon Sources Switching

**Figure 5:**
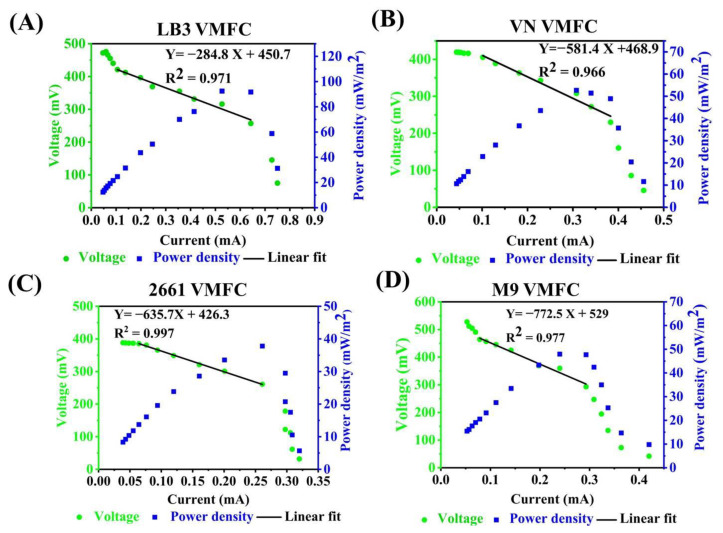
The performance evaluation of VMFCs with media switching. Polarization and power density curves obtained by varying the external resistance over a range of 100 to 9999 Ω from LB3 VMFC (**A**), VN VMFC (**B**), 2661 VMFC (**C**), and M9 VMFC (**D**).

Next, we evaluated the performance of VMFC with different media. Three reported media (LB3 medium [26], M9 medium [26], and VN medium [29]) and a 2661 medium were selected to test the effects of medium switching on the output voltages, apparent internal resistance, and power density of VMFC, respectively. To calculate the apparent internal resistance, the polarization curves were plotted based on the steady-state discharge method as follows. The external circuit voltages (U) were recorded by varying the external circuit resistance of adjustable resistor (R, 100, 200, 400, 600, 800, 1000, 1500, 2000, 3000, 4000, 5000, 6000, 7000, 8000, and 9999 Ω) and kept for 300 s for the external circuit voltage to reach a steady state. The current was obtained from I = U/R, and the polarization curve was plotted with current as the horizontal coordinate and voltage as the vertical coordinate. The polarization curves can be roughly divided into three stages: activation polarization, ohmic loss, and concentration polarization. In the ohmic loss, the external output power of MFCs is the highest, and the polarization curve is linear. The apparent internal resistance of MFC is equivalent to the slope of the polarization curve obtained by fitting the data of the polarization curve in the ohmic loss [46]. As shown in Figure 5 and Table 1, LB3 MFC had the most outstanding performance with the lowest apparent internal resistance (284.8 ± 13.7 Ω), and highest output power (Table 1, 91.1 ± 4.5 mW/m^2^). In addition, the growth rate of *V. natriegens* in M9 MFC, VN MFC, and 2661 MFC was calculated as 0.07 h^−1^, 0.05 h^−1^ and 0.06 h^−1^, which is lower than in the LB3 MFC (0.08 h^−1^) (Figure S1). These results indicated that *V. natriegens* is able to assimilate different media to produce bioelectricity in MFC. Comparing the four kinds of VMFCs, we found that LB3 MFC had the most outstanding performance with the minimum apparent internal resistance and maximum power density. In addition, notably, the concentration of sodium chloride used in the LB3 medium is 30 g/L, suggesting *V. natriegens* in MFC is able to assimilate high-salinity culture substrates to generate bioelectricity. Therefore, we further evaluated the higher salinity tolerance ability of VMFC using LB6 medium (LB6 VMFC, LB medium supplemented with 50 g/L sodium chloride, the total concentration of sodium chloride in LB6 is 60 g/L). As shown in Table 1, the VMFC using LB6 medium delivered satisfactory maximum power 108.6 ± 2.9 mW/m^2^, indicating that the VMFC has an excellent ability of high salinity tolerance and bioelectricity generation simultaneously.

We further substituted glucose, the sole carbon source in VN MFC, with eight other carbon sources to evaluate the effects of carbon sources switching on the output power of VMFC. As shown in Table 1, all eight electronic mediators tested were also able to generate bioelectricity. Among them, using 1% glycerol as a carbon source in VMFC delivered the highest output power (111.9 ± 6.7 mW/m^2^), which is higher than the VMFC used glucose as a carbon source (91.1 ± 4.5 mW/m^2^).

### 3.4. The Insight of the Endogenous Electronic Mediator Biosynthesis of V. natriegens

As a feedstock flexibility and high salt-tolerant electricity-producing microorganism, the deficiency of endogenous electron mediator biosynthesis capacity of *V. natriegens* limits the application of VMFCs; therefore, we also gave insight into the endogenous electron mediator’s biosynthesis of *V. natriegens* to provide guidance for enhancing the biosynthesis capacity of endogenous electronic mediators in VMFC.

**Figure 6:**
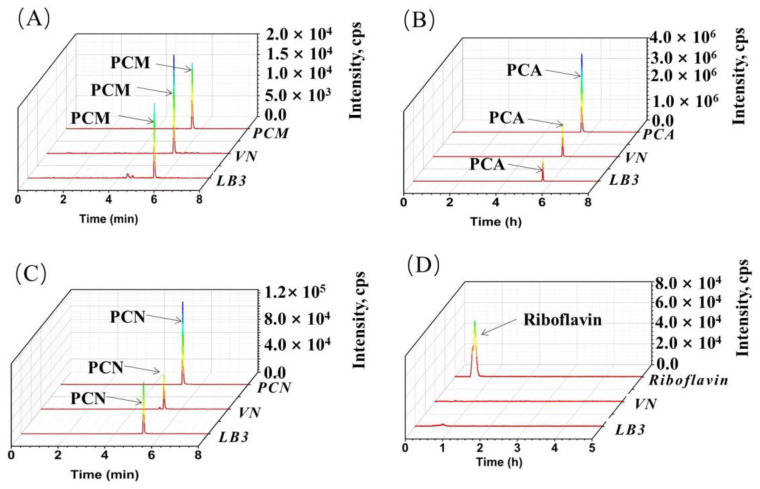
LC-MS chromatograms (extracted ion chromatograms in positive mode) of PCM (**A**), PCA (**B**), PHZ (**C**), and riboflavin (**D**) in LB3 MFC and VN MFC media. Retention time for the PCA, PCM, HPZ, and riboflavin are 5.61, 5.23, 5.59, and 0.54 min, respectively. PCA, phenazine-1-carboxylic acid; PCM, phenazine-1-carboxamide; HPZ, 1-hydroxyphenazine; VN, VN medium; LB, LB medium.

We first characterized four potential physiological electron mediators—PCM, PCA, PHZ, and riboflavin by LC-MS/MS in VMFC with LB3 and VN media, the two most widely used media for culturing *V. natriegens* at present. The mass-to-charge ratios (*m*/*z*) of the main ion fragment of riboflavin provided by AB Sciex Instruments is 377.1 (parent ion) → 243.1 (daughter ion), and the parent ions and daughter ions of PCA, PCM, and HPZ standard obtained in this study were 225.1 → 179.0/207.1, 224.1 → 179.1/152.1 and 197.1 → 179.1/169.1, respectively. The four physiological electron mediators were analyzed by parent ion and daughter ion in the scanning mode of MRM. As shown in Figure 6, PCA, PCM, and HPZ were found in both MFCs with the concentration of 7.4 ± 0.16, 9.1 ± 0.2, 3.2 ± 0.02, and 22.9 ± 0.7 μg/L in LB3 MFC, and the concentration of 12.0 ± 0.17, 5.1 ± 0.08 and 3.9 ± 0.07 μg/L in VN MFC respectively, but not in VN and LB3 media not inoculated with *V. natriegens* in MFCs (control group, data not shown). However, riboflavin, an efficient electron mediator secreted by electricity-producing microorganisms with the parent ions and daughter ions of 377.1 → 243.1, was not detected in both MFCs (data not shown).

**Figure 7:**
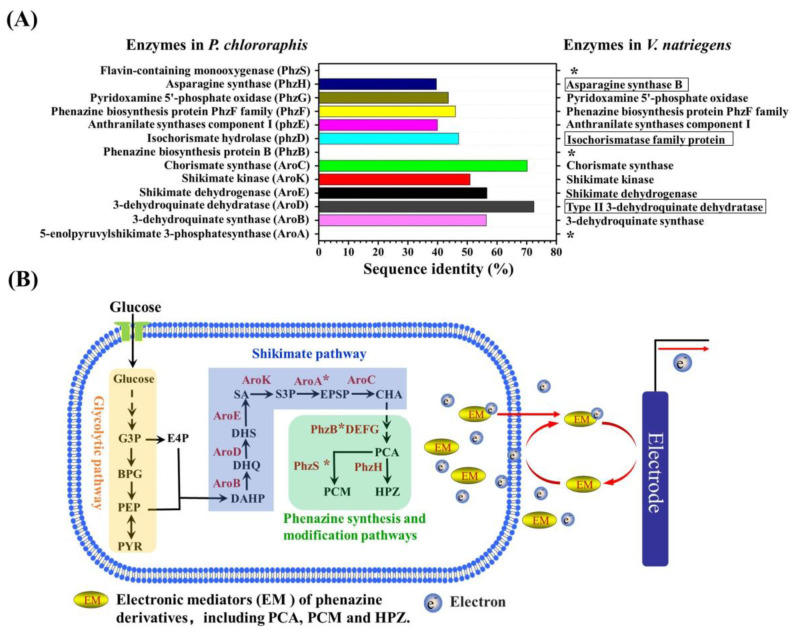
Protein sequence alignment and an identified biosynthesis pathway of phenazine derivatives electron mediators in *V. natriegens*. (**A**) Protein sequence alignment of phenazine derivatives electron mediators in *V. natriegens* by using the reported protein sequences of phenazine derivatives from *P. chlororaphis* and BLAST. Left axis: the names of enzymes reported in *P. chlororaphis* for the biosynthesis of phenazine derivatives; right axis: the names of enzymes identified in *V. natriegens* by BLAST. The mark of “*” denotes that the corresponding enzyme is not found in *V. natriegens* by protein sequences alignment. These enzymes in the rectangular box indicate that their names marked in the protein sequences of *V. natriegens* are different from that in *P. chlororaphis*. (**B**) The identified biosynthesis pathway of phenazine derivatives electron mediators in *V. natriegens* based on the LC-MS/MS characterization and protein–protein sequence alignment, as well as previous reports. G3P: glyceraldehde-3-phosphate; BPG: 1,3-bisphosphoglycerate; PEP: phosphoenolpyruvate; E4P: Erythrose 4-phosphate; DAHP: 3-deoxy-D-arabino-heptulosonate-7-phosphate; PYR: pyruvate; DHQ: 3-dehydroquinate; DHS: 3-dehydroshikimate; SA: shikimate; S3P: shikimate-3-phosphate; EPSP: 5-enolpyruvylshikimate 3-phosphate; CHA: chorismate; PCA: 1-carboxylic acid, PCM: phenazine-1-carboxamide; HPZ:1-hydroxyphenazine; AroB: 3-dehydroquinate synthase; AroD: 3-dehydroquinate dehydratase; AroE: shikimate dehydrogenase; AroK: shikimate kinase; AroA: 5-enolpyruvylshikimate 3-phosphate (EPSP) synthase; AroC: chorismate synthase; PhzB?DEFG: the abbreviations of five enzymes for PCA synthesis encoded by the same gene cluster, and the five enzymes are PhzB (phenazine biosynthesis protein B), phzD (isochorismate hydrolase), phzE (anthranilate synthases component I), PhzF (phenazine biosynthesis protein PhzF family) and PhzG (pyridoxamine 5’-phosphate oxidase), respectively. The mark of “*” denotes that the corresponding enzyme is not found in *V. natriegens* by protein sequence alignment. PhzS: flavin-containing monooxygenase, PhzH: asparagine synthase.

Further, we identified the biosynthesis pathway of PCM, PCA, and PHZ in VMFC. Since there are no relevant reports on the above-mentioned electron mediator synthesis pathways in *V. natriegens*, we compared the protein sequences of *Vibrio natriegens* NBRC 15,636 = ATCC 14,048 = DSM 759 (taxid:1219067) from the National Center for Biotechnology Information (NCBI) with reported protein sequences for synthesizing PCM, PCA, and PHZ in *Pseudomonas chlororaphis* by protein–protein alignment using protein–protein Basic Local Alignment Search Tool (BLAST). The PCM, PCA, and PHZ are phenazine derivatives [47,48]. In the reported synthesis pathway of phenazine derivatives, the first substrate in the core biosynthetic pathway for the synthesis of strain-specific phenazines is considered to be the chorismic acid from the shikimate pathway, initiated by erythrose 4-phosphate and phosphoenolpyruvate [49,50]. The synthesis of chorismic acid by the shikimate pathway mainly encompasses six enzymes: 3-dehydroquinate synthase (AroB), 3-dehydroquinate dehydratase (AroD), shikimate dehydrogenase (AroE/YdiB), shikimate kinase (AroK/AroL), 5-enolpyruvylshikimate 3-phosphate synthase (AroA), and chorismate synthase (AroC). We used these protein sequences of six enzymes from *Pseudomonas chlororaphis* as the reference sequences to align with the protein sequences from *V. natriegens* by BLAST. As shown in Figure 7A, five of these six enzymes were found as the following: AroB, AroD, AroE, AroK, and AroC except AroA with the sequence identity of 56.39%, 72.41%, 56.62%, 50.94%, and 70.19%, respectively. Among them, the AroD (3-dehydroquinate dehydratase) was marked as “type II 3-dehydroquinate dehydratase”. These results indicate that chorismic acid can be synthesized by shikimate pathway in *V. natriegens*. According to previous reports [47,48,51], chorismic acid is next metabolized to synthesize PCA and phenazine-1,6-dicarboxylic acid (PDC) through five enzymes: phenazine biosynthesis protein B (PhzB), isochorismate hydrolase (PhzD), anthranilate synthases component I (PhzE), phenazine biosynthesis protein PhzF family (PhzF), and pyridoxamine 5’-phosphate oxidase (PhzG). Finally, PCA is modified by flavin-containing monooxygenase (PhzS) and asparagine synthase (PhzH) to synthesize PCM and HPZ, respectively. In order to find the metabolic pathway from chorismic acid to PCA, PCM, and HPZ, similarly, we compared these protein sequences from *Pseudomonas chlororaphis* with the protein sequence of *V. natriegens* by BLAST. We found 47.09%, 39.93%, 46.01%, 43.60%, and 39.60% sequence identity for phzD, phzE, PhzF, PhzG, and PhzH, respectively, in *V. natriegens*. Among them, PhzD (isochorismate hydrolase) and PhzH (asparagine synthase) were labeled as “isochorismatase family protein” and “asparagine synthase B”, respectively. Although the sequence of PhzB was not found, PhzB, phzD, phzE, PhzF, and PhzG are encoded by the gene cluster of *phzBDEFG* [47,48]. Therefore, we identified that the synthesis of PCA by branched acid is catalyzed by these enzymes of PhzB, PhzD, PhzE, PhzF, and PhzG, and then the synthetic PCA is further, respectively, modified by PhzH and PhzS to synthesize HPZ and PCM in *V. natriegens*. Summarizing the results of protein–protein sequence alignment, we herein condensed the biosynthesis pathway of phenazine derivatives electron mediators in VMFC. As shown in Figure 7B, the biosynthesis pathways of phenazine derivatives of *V. natriegens* start from chorismic acid synthesized by the shikimate pathway, then the chorismic acid is metabolized to synthesize PCA by phenazine synthesis pathway, and finally, the PCA is further modified through phenazine modification pathway to synthesize HPZ and PCM. The synthesized PCA, HPZ, and PCM are then used to transfer electrons secreted by the cells to the anode in VMFC. The biosynthetic pathways identification of PCM, PCA, and PHZ in VMFC laid a solid foundation for guiding the biosynthesis regulation of electron mediators by metabolic engineering to improve the performance of VMFC.

## 4. Discussion

In the absence of adding electron mediators, VMFC delivered an external circuit voltage of 20~30 mV, and no biofilm formation and cell adhesion were found on the surface of the carbon cloth anode (Figure 3). In light of this and the previous reports, we infer that the extracellular electron transfer of *V. natriegens* is mediated electron transfer and requires the addition of exogenous electron mediators to enhance the electron transfer capability of VMFC. Seven physiological or non-physiological electron mediators were then applied to VMFC (Table 1), and the results showed that all seven electronic mediators tested were able to enhance the output powers of MFCs, which is similar to the industrial microorganisms applied to MCF, such as *E. coli* and *S. cerevisiae*. For instance, PARK [52] and Rong Xie [53] used exogenous neutral red as an electron mediator to increase the bioelectricity generation in MFC, respectively. Those results revealed that *V. natriegens* is electroactive and are capable of being used to generate bioelectricity in MFCs with the addition of various electron mediators, which, to the best of our knowledge, have not been described previously.

When the electronic mediator MB was added, both the external circuit voltage and the open circuit voltage of VMFC were higher than MFCs with *E. coli* and *S. cerevisiae*. The stable external circuit voltages of VMFC are between approximately 300 and 400 mV, which is similar to the *E. coli* and *S. cerevisiae* MFCs [9,52,54]. Comparing the maximum power densities of VMFC with *E. coli* MFC (41.1 ± 3.5 mW/cm^2^) and *S. cerevisiae* MFC (47.4 ± 3.3 mW/cm^2^) in Table 1, the maximum power densities of some VMFCs, such as VMFC used glycerol (111.9 ± 6.7 mW/cm^2^) or sucrose (99.1 ± 4.8 mW/cm^2^) as a carbon source, were higher than those of *E. coli* and *S. cerevisiae* MFC. Furthermore, the maximum power density of 120.33 mW/cm^2^ reported by Nandy [55] for nonrecombinant *E. coli* was similar to the maximum power density in our study using glycerol as a carbon source. It can therefore be concluded that, similar to *E. coli* and *S. cerevisiae*, *V. natriegens* also has promising application prospects in terms of bioelectricity production. In this study, the external circuit voltage of VMFC is significantly low when the absence of electron mediators, suggesting that *V. natriegens* cannot transfer more electrons to the anode via endogenous electron mediators. The biosynthesis of endogenous electronic mediators PCA, HPZ, and PCM were also identified via the shikimate pathway and phenazine modification pathway (Figure 7B). However, according to Table 1, the addition of PCA, HPZ, and PCM alone in VMFC does not effectively improve the ability of electron transfer. Therefore, in order to enhance the electron transfer efficiency and reduce the use of exogenous electron mediators of VMFC, modulating the shikimate pathway and phenazine modification pathway to attempt simultaneously boost the biosynthesis of PCA, HPZ, and PCM is one possible approach in further studies. In addition, notably, riboflavin, a commonly known efficient redox mediator, was not detected in both VMFCs. Therefore, to heterogeneously express the riboflavin biosynthesis pathway in *V. natriegens* and improve the riboflavin biosynthesis of *V. natriegens* by metabolic engineering may also be an effective approach to boost electron transfer of VMFC, and further study remains to be elucidated.

It has been reported that *V. natriegens* can assimilate different carbon sources for growth (“feedstock flexibility”) [28,29]. Herein, we tested four media and nine carbon sources for VMFCs, and the results indicated that *V. natriegens* is able to metabolize different media and carbon sources to produce bioelectricity in MFC. Among them, six carbon sources used were able to generate an output power over 50 mW/cm^2^ in VMFC, illustrating that VMFCs also have “feedstock flexibility”. In addition, notably, all reported electroactive industrial microorganisms, including *E. coli* and *S. cerevisiae,* etc., are unable to tolerate high salinity stress, hindering the development and application of MFC using industrial microorganisms for high salinity substrates. However, herein, the VMFC delivered maximum power 111.9 ± 6.7 mW/cm^2^ with LB6 high salt medium (containing 60 g/L NaCl), indicating that the VMFC has an excellent ability of high salinity tolerance and bioelectricity generation simultaneously. In light of this, considering the feedstock flexibility, high salinity tolerance ability, and high bioelectricity generation ability of VMFC, it is reasonable to refer that VMFC can be promisingly employed in the biodegradation of complex organic substances from high salinity wastewater such as seafood processing produces wastewater (characterized by salinity between 30 and 50 g/L NaCl) [21] and high-salinity textile wastewater (characterized by salinity between 50 and 60 g/L NaCl) [22] for the treatment of wastewater and energy harvesting simultaneously, and the further study remains to be elucidated.

According to previous reports [30,32], *V. natriegens* is experiencing rapid growth. However, in this study, we found that the growth of the cells is relatively slow in MFC, which may be based on two reasons; one is that the anode of MFC for cell growth is anaerobic, which results in slow cell growth due to lack of sufficient oxygen, and the other is that the initial inoculum (OD = 1.7) of *V. natriegens* for running MFC is relatively high, and the high initial cell density also inhibits the cell growth in MFC.

By characterizing the potential physiological electron mediators using LC-MS/MS, three phenazines derivatives electron mediators—PCM, PCA, and PHZ—were found in VMFC, but at low levels (Figure 6), which is consistent with the previous speculation that *V. natriegens* can synthesize endogenous electron mediators but are deficient in the MFCs. Further, the biosynthesis pathway of PCM, PCA, and PHZ was identified to be via the shikimate pathway and the phenazine synthesis and modification pathways in *V. natriegens* by protein–protein alignment, laying a solid foundation for guiding the biosynthesis regulation of electron mediators by metabolic engineering to further improve the performance of VMFC.

## 5. Conclusions

Herein, a novel application of *V. natriegens* as an electroactive microorganism for MFC was studied. The extracellular electron transfer of VMFC is mediated electron transfer, and seven electronic mediators texted can improve the electron transfer capability of VMFCs. VMFC using different media and carbon sources delivered satisfactory output power density, revealing that VMFCs have excellent bioelectricity generation performance, feedstock flexibility, and high salt resistance. Three phenazine derivatives electron mediators (PCM, PCA, and PHZ) were found in VMFC, and their biosynthesis pathways were identified to be via the shikimate pathway and phenazine synthesis and modification pathways by protein–protein sequence alignment. This work provided a novel high salt-tolerant and feedstock flexibility electroactive microorganism for MFC and gave insight into the endogenous electron mediator biosynthesis of *V. natriegens*, which is of great value as a baseline for meaningfully expanding the application of *V. natriegens* to MFC and even EF.

## Figures and Tables

**Table 1:** The maximum power density of MFCs with electron mediators, media, and carbon sources switching.

Electron Mediators	Media	Maximum Power Density (mW/cm^2^)	Carbon Sources	Mediators	Maximum Power Density (mW/cm^2^)
MB (50 mg/L)	LB3	91.1 ± 4.5	Glucose (10 g/L)	MB	71.9 ± 6.3
Riboflavin (40 mg/L)	LB3	0.9 ± 0.3	Glycerol (10 g/L)	MB	111.9 ± 6.7
PCA (20 mg/L)	LB3	8.0 ± 0.2	SA (2.5 g/L)	MB	81.6 ± 3.3
PCM (20 mg/L)	LB3	3.2 ± 0.2	PG (2.5 g/L)	MB	46.4 ± 2.1
HPZ (60 mg/L)	LB3	2.4 ± 0.1	Sucrose (10 g/L)	MB	99.1 ± 4.8
DAD (41 mg/L)	LB3	1.8 ± 0.1	FA (2.5 g/L)	MB	22.4 ± 1.2
HA (1.3 g/L)	LB3	2.3 ± 0.1	Fructose (10 g/L)	MB	85.5 ± 3.9
**Media**	**Mediator**	**Maximum Power Density (mW/cm^2^)**	ME (10 g/L)	MB	75.1 ± 2.8
			Arabinose (10 g/L)	MB	31.0 ± 2.0
LB3	MB	91.1 ± 8.5	**Media**	**Mediator**	**Maximum Power Density (mW/cm^2^)**
M9	MB	56.4 ± 3.3			
VN	MB	71.9 ± 3.8	LB	MB	41.1 ± 3.5
2661	MB	40.4 ± 2.5	YPD	MB	47.4 ± 3.3
LB6	MB	108.6 ± 2.9			

The data represented on the orange background are the maximum power density of VMFCs, and the data represented on the blue background are the maximum power density of *E. coli* MFC (using LB medium) and *S. cerevisiae* MFC (using YPD medium).

## Data Availability

All datasets generated for this study are included in the article/Supplementary Materials.

## Supplementary Materials

The following supporting information can be downloaded at: https://www.mdpi.com/article/10.3390/microorganisms11020490/s1, Figure S1: The growth of *V. natriegens* in LB3 MCF, VN MCF, M9 MCF, and 2661 MCF.
