# Supplementary material for: Identification of Emerging Industrial Biotechnology Chassis Vibrio natriegens as a Novel High Salt-Tolerant and Feedstock Flexibility Electroactive Microorganism for Microbial Fuel Cell
Source: Microorganisms. 2023 Feb 16;11(2):490. doi: 10.3390/microorganisms11020490 (PMC9961702; doi:10.3390/microorganisms11020490)
Supplement: Supplementary file 1 [file microorganisms-11-00490-s001.zip › microorganisms-2151598-supplementary.pdf]

Identification of Emerging Industrial Biotechnology Chassis *Vibrio natriegens* as a Novel High Salt-Tolerant and Feedstock Flexibility Electroactive Microorganism for Microbial Fuel Cell

Zhijin Gong [1,2], Rong Xie [1,2], Yang Zhang [1,2], Meng Wang [1,2] and Tianwei Tan [1,2,*]

[1] National Energy R&D Center for Biorefinery, College of Life Science and Technology, Beijing University of Chemical Technology, Beijing 100029, China
[2] Beijing Key Laboratory of Bioprocess, College of Life Science and Technology, Beijing University of Chemical Technology, Beijing 100029, China
* Correspondence: biorefinery@mail.buct.edu.cn

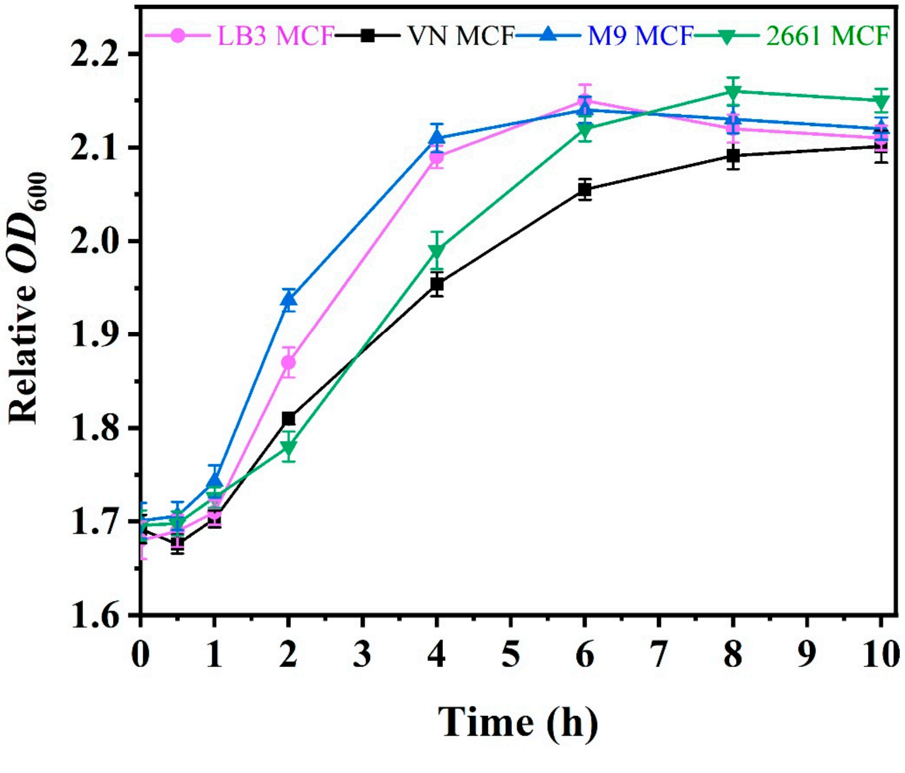

Figure S1: The growth of *V. natriegens* in LB3 MCF, VN MCF, M9 MCF and 2661 MCF.
